# Anti-ribosomal P protein IgG autoantibodies in patients with systemic lupus erythematosus: diagnostic performance and clinical profile

**DOI:** 10.1186/1741-7015-11-98

**Published:** 2013-04-04

**Authors:** Diana Carmona-Fernandes, Maria José Santos, Helena Canhão, João Eurico Fonseca

**Affiliations:** 1 Rheumatology Research Unit, Instituto de Medicina Molecular, Lisbon Academic Medical Centre, Av. Prof. Egas Moniz, Edifício Egas Moniz, Lisboa, 1649-028, Portugal; 2 Rheumatology Department, Hospital Garcia de Orta, Av. Torrado da Silva, Pragal, Almada, 2801-951, Portugal; 3 Rheumatology and Bone Metabolic Diseases Department, Hospital de Santa Maria, Av. Prof. Egas Moniz, Lisboa, 1649-035, Portugal

**Keywords:** Anti-Rib-P, Systemic lupus erythematosus, Antibodies

## Abstract

**Background:**

This study was devised to assess the performance of anti-ribosomal P (anti-Rib-P) antibodies in the diagnosis of systemic lupus erythematosus (SLE) and the association of these antibodies with the clinical features of SLE.

**Methods:**

We used a fluorescence enzyme immunoassay to determine anti-Rib-P levels in an SLE group, a rheumatic disease control (RDC) group (rheumatoid arthritis (RA), ankylosing spondylitis, psoriatic arthritis and juvenile idiopathic arthritis), and a healthy control (HC) group. We also determined anti-Smith antigen (anti-Sm) and anti-double-stranded DNA (anti-dsDNA) antibody levels. Receiver operating characteristic (ROC) curves were constructed and the best cut-off points for positivity were determined. Using regression analysis, the relationship between clinical variables and autoantibody levels was analyzed.

**Results:**

In total, 127 patients with SLE, 256 controls with other rheumatic diseases, and 100 HCs were studied. Anti-Rib-P autoantibodies were positive in 18 (14.2%) of the patients with SLE (mean concentration of 30.6 ± 46.9 U/ml) and in 2 patients with RA (0.8% of the RDC group). In addition, 12 patients with SLE (9.4%) were positive for anti-Sm (31.1 ± 40.8 U/ml) and 63 (49.6%) were positive for anti-dsDNA autoantibodies (88.4 ± 88.5 U/ml). When we assessed the 18 patients with SLE who had tested positive for anti-Rib-P, we found that 4 of these were positive for anti-Rib-P only, whereas 12 were positive for anti-Rib-P plus anti-dsDNA, and 2 were positive for all three antibodies. There were no samples positive for anti-Rib-P plus anti-Sm. The specificity, sensitivity, positive likelihood ratio, and negative likelihood ratio of anti-Rib-P for SLE diagnosis were 99.4%, 14.2%, 23.7%, and 0.86%, respectively.

Caucasian ethnicity was associated with lower anti-Rib-P antibody levels. No relation was found between anti-Rib-P levels and neuropsychiatric or other clinical features.

**Conclusions:**

Anti-Rib-P autoantibodies have high specificity for SLE, and measurement of these might improve the accuracy of SLE diagnosis. In this study, we found that Caucasian ethnicity was associated with lower anti-Rib-P antibody levels.

## Background

Systemic lupus erythematosus (SLE) is a chronic autoimmune disease characterized by multi-organ involvement and by the production of autoantibodies directed against a variety of nuclear and cytoplasmic antigens [1,2]. Autoantibodies can be detected in patients’ sera years before the diagnosis of SLE is made [3]. Some antibodies are relevant to diagnosis, whereas others are associated with prognostic features or disease activity status [2,4].

Antibodies against double-stranded DNA (anti-dsDNA) and Smith antigen (anti-Sm) are considered very specific for SLE diagnosis, and both are part of the immunologic classification criteria for this disease [5]. Furthermore, high levels of anti-dsDNA are associated with higher disease activity in SLE [6].

One subset of SLE-specific autoantibodies is directed against ribosomal P (Rib-P) phosphoproteins [2]. The Rib-P antigen consists of three protein components of the 60S ribosomal subunit designated P0 (38 kDa), P1 (19 kDa), and P2 (17 kDa). A pentameric complex of one copy of P0 and two copies each of P1 and P2 interacts with the 28S rRNA molecule to form a GTPase domain that is active during the elongation step of protein translation [7-12]. The major immunoreactive epitope of these ribosomal antigens has been localized to the 22 amino acid carboxy-terminal domain, which is present in all three proteins, and contains two phosphorylated serine residues proteins [2,8-14].

Anti-Rib-P antibodies are directed against the three subunits [2,9,15], and are able to penetrate certain cells, binding to ribosomal proteins and blocking protein synthesis [15]. Anti-Rib-P antibodies enhance the production of tumor necrosis factor (TNF) and interleukin (IL)-6 by activated monocytes and also upregulate the expression of TNF and IL-6 messenger RNA in activated monocytes, indicating that human peripheral blood monocytes express the ribosomal P epitope upon activation [15].

Ethnic background may influence the likelihood of anti-Rib-P antibodies occurring in patients with SLE, with the frequency ranging from 6% to 46% in different ethnic groups [2,7,11,14-16]. In most ethnic groups, anti-Rib-P antibodies are present in 6 to 20% of patients, whereas 36% of Chinese patients with SLE were reported to be positive [7,11,12,15].

Anti-Rib-P antibodies seem to be highly specific for SLE, and might also be a marker for SLE disease activity [12,14,15]. The presence of anti-Rib-P antibodies in patients with SLE has been reported to be associated with younger age at disease onset, multiple organ involvement, and an overall severe disease course [8], including presence of central nervous system involvement [2,4,7,11,12,15], nephritis [2,7,12,15], photosensitivity [2], malar rash [2], and hepatic involvement [2,7,12]. Moreover, it has become evident that anti-Rib-P antibodies are more prevalent in juvenile-onset than in adult onset SLE [11,12]. Bonfa *et al*. first assessed the association of anti-Rib-P antibodies with psychiatric features in patients with psychosis secondary to SLE [17]; however, other studies have not confirmed this association [7,8].

We hypothesized that anti-Rib-P autoantibodies might be useful for SLE diagnosis. To test this hypothesis, we used a new fluorescence enzyme immunoassay (FEIA) kit to quantify levels of anti-Rib-P in patients with SLE, controls with other rheumatic diseases (rheumatic disease control (RDC) group, which included rheumatoid arthritis (RA), juvenile idiopathic arthritis (JIA), ankylosing spondylitis (AS), and psoriatic arthritis (PsA)), and healthy controls (HC group).

## Methods

### Ethics approval

The study was conducted in accordance with the regulations governing clinical trials such as the Declaration of Helsinki, as amended in Seoul (2008), and was approved by the ethics committees of the Centro Hospitalar Lisboa Norte, Hospital de Santa Maria and the Hospital Garcia de Orta. All participants signed a written informed consent form before any protocol-specific procedure was carried out.

### Patients

For this study, we used serum samples from Biobank (Instituto de Medicina Molecular, Lisboa, Portugal), collected between May 2007 and December 2009. Samples were selected from patients with the following diagnoses fulfilling the criteria of the relevant classifications: SLE (revised American College of Rheumatology (ACR) criteria, 1997), RA (revised American Rheumatism Association (ARA) criteria, 1987), JIA (International League of Associations for Rheumatology (ILAR) classification, second revision, 2001), AS (modified New York criteria, 1984) and PsA (modified European Spondyloarthropathy Study Group (ESSG) criteria, 2006). Samples from healthy volunteers were used as the HC group.

In total 127 patients with SLE, 256 RDCs (100 RA, 99 AS, 34 JIA, and 23 PsA), and 100 HCs were studied. Data on age, ethnicity, and gender were collected. For patients with SLE, the following data were obtained at the time of blood sample collection: age at disease diagnosis, disease duration, cumulative clinical features (in accordance with ACR classification criteria), presence of autoantibodies (anti-dsDNA, anti-Sm, anti-cardiolipin, anti-SSA, anti-SSB, and anti-ribonucleoprotein (anti-RNP) antibodies), current medication (including current dosage of corticosteroids and use of immunosuppressants, current disease activity (evaluated using the Systemic Lupus Erythematosus Disease Activity Index 2000 (SLEDAI2K) [18]), and cumulative organ damage (scored using the Systemic Lupus International Collaborating Clinics/ACR Damage Index (SLICC) [19]). Clinical features, particularly the occurrence of neuropsychiatric lupus syndromes in accordance with the ACR nomenclature [20], disease activity, and accumulated organ damage were assessed semi-annually thereafter.

### Assay

Quantification of anti-Rib-P, anti-Sm and anti-dsDNA antibodies was carried out using FEIA kits (EliA™ Rib-P, EliA™ Sm, and EliA™ dsDNA; Phadia, Uppsala, Sweden; now part of Thermo Fisher Scientific) for *in vitro* diagnosis in accordance with the manufacturer’s instructions.

### Statistical analysis

Results are reported as mean ± standard deviation for continuous variables or proportion for categorical variables. Anti-Rib-P, anti-Sm and anti-dsDNA concentrations are presented in U/ml.

Receiver operating characteristic (ROC) curves were performed for each test comparing the results from the patients with SLE with those of the HC or RDC groups. For both ROC curves for each antibody, a cut-off point was determined as the value of the parameter corresponding to the highest sensitivity without lowering the specificity. The area under the curve (AUC) was also determined.

Differences between the SLE and control groups were assessed using the *t*-test for continuous variables or χ^2^ or Fisher’s exact test for proportions.

The association between clinical variables and the various antibodies was investigated for patients with SLE using univariate followed by multivariate linear regression analyses. All variables relating to the studied outcome in the univariate analyses at *P* ≤ 0.20 were considered potential predictors, and were entered into the multivariate linear regression models along with neuropsychiatric features, because of their previously described association with these antibodies. The selection of covariates was stepwise by backward selection.

Statistical calculations were performed using SPSS statistical software (version 15.0; SPSS Inc., Chicago, IL, USA) and a two-tailed *P*-value of < 0.05 was considered significant.

## Results

The demographic characteristics of all studied subjects are presented in Table 1. Patients with SLE had a mean age at disease diagnosis of 34.2 ± 14.5 years, disease duration of 8.3 ± 6.5 years (range 0.5 to 34 years), mean SLEDAI2K of 3.3 ± 4.2, and SLICC damage score of 1.1 ± 2.1 at baseline evaluation. Of the 127 patients with SLE, 79 (62.2%) were receiving treatment with corticosteroids (mean daily dosage of prednisolone 12.4 mg), 90 (71%) with antimalarials, 50 (39%) with immunosuppressants, and 1 with a monoclonal antibody (rituximab).

**Table 1:** Demographic characteristics of the studied population

	**SLE**	**HC**	**Rheumatic disease control**			
			**RA**	**JIA**	**AS**	**PsA**
Age, years	43.6 ± 14.1	43.1 ± 14.6	51.7 ± 13.9	13.6 ± 7.2	41.9 ± 10.7	49.8 ± 11.1
Females, n (%)	120 (96.0)	91 (91.9)	99 (99.0)	18 (52.9)	26 (27.1)	9 (39.1)
Caucasian, n (%)	110 (88.0)	98 (99.0)	90 (90.0)	30 (88.2)	95 (97.9)	20 (90.9)

Abbreviations: AS, ankylosing spondylitis; HC, healthy control; JIA, juvenile idiopathic arthritis; PsA, psoriatic arthritis; RA, rheumatoid arthritis; SLE, systemic lupus erythematosus. Values represent mean ± standard deviation for years, and proportions for categorical variables.

**Figure 1:**
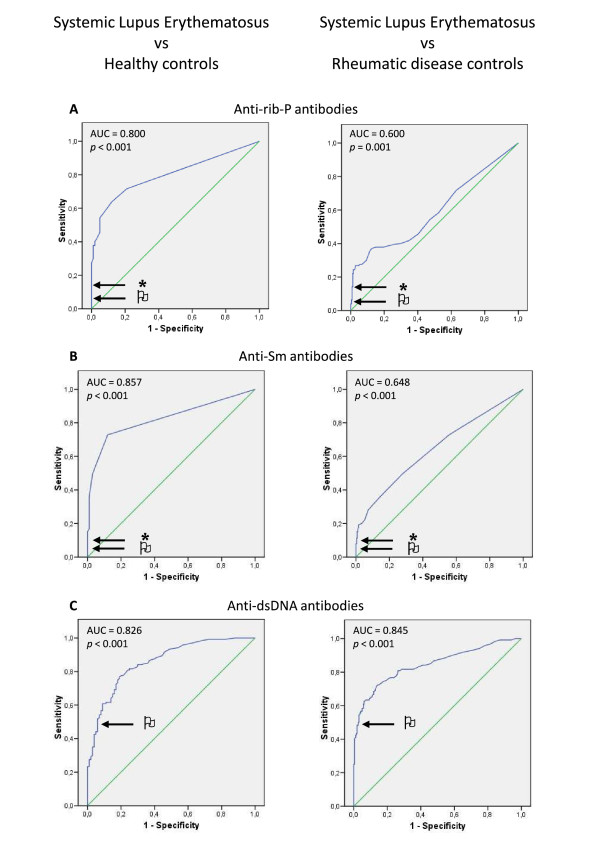
**Receiver operating characteristic (ROC) curves for the three antibodies quantified.** (**A**) Anti-ribosomal P (anti-Rib-P), (**B**) anti-Smith (anti-Sm), and (**C**) anti-double-stranded DNA (anti-dsDNA) antibodies. The curves represent the sensitivity and specificity for the systemic lupus erythematosus (SLE) group versus the healthy control group, and for the SLE group versus the rheumatic disease control group. For each curve, the area under the curve (AUC) and corresponding *P*-value are indicated. The flag indicates the cut-off established by the manufacturer and the asterisk indicates the new cut-off calculated from the curves (4.45 U/ml for anti-Rib-P and 3.4 U/ml for anti-Sm antibodies; the anti-dsDNA cut-off did not change).

ROC curves were constructed to obtain the most adequate cut-off values for the Portuguese population; these curves are of particular relevance for the new anti-Rib-P test kit. Curves were also constructed for the other tests for coherence analysis. The curves are presented in Figure 1 with the AUC and the corresponding *P*-values identified. For both the anti-Rib-P and anti-Sm tests, the cut-off values after analysis of the ROC curves were 4.45 U/ml and 3.4 U/ml, respectively. For anti-dsDNA, the cut-off value given by the manufacturer (15 U/ml) was used because it corresponds to the value obtained from the ROC curves. With these adjustment in cut-off values, we identified a higher number of patients with SLE who were positive for either anti-Rib-P or anti-Sm, without incurring more false-positive results in the control groups than we did with the manufacturer cut-off points (data not shown).

We found that the levels of anti-Rib-P autoantibodies were significantly higher in the SLE group (mean concentration of 4.9 ± 20.2 U/ml) than in the HC group (0.07 ± 0.21 U/ml; *P =* 0.016) or the RDC group (0.6 ± 1.8 U/ml; *P =* 0.017). In 18 samples of the SLE group (14.2%), anti-Rib-P was above the cut-off value of 4.45 U/ml for positivity (mean concentration of 30.6 ± 46.9 U/ml). Of note, in the RDC group, two patients with RA (0.8%) were positive for anti-Rib-P autoantibodies (18.9 ± 9.8 U/ml), whereas none of the HCs tested positive for this antibody.

The mean concentration of anti-Sm antibodies for the whole SLE group was 2.8 ± 13.8 U/ml, and 12 of these positive samples (9.4%) had a significantly higher mean concentration (31.1 ± 40.8 U/ml) than those of the HC group (0.02 ± 0.11 U/ml; *P =* 0.028) or the RDC group (0.1 ± 0.3 U/ml; *P =* 0.035). Positive results (above 3.4 U/ml) for anti-Sm autoantibodies were found only in the SLE group.

Patients with SLE also had a significantly greater mean concentration of anti-dsDNA antibodies (44.6 ± 73.8 U/ml) than found in the HC group (3.5 ± 8.1 U/ml; *P* < 0.001) or the RDC group (2.6 ± 4.2 U/ml; *P* < 0.001). Of the 127 patients with SLE, 63 (49.6%) were positive for anti-dsDNA (mean concentration 88.4 ± 88.5 U/ml.), compared with 6 samples (6.0%) in the HC group and 5 samples (2.0%) in the RDC group (1 RA, 1 JIA, and 3 AS samples).

These results are summarized in Table 2. The performance of the tests was evaluated, for sensitivity, specificity, positive likelihood ratio (LR^+^) and negative likelihood ratio (LR^−^), and found to be 14.2%, 99.4%, 23.7, and 0.86, respectively, for anti-rib-P; 9.4% 100%, ∞, and 0.90, respectively, for anti-Sm ; and 49.6%, 96.9%, 16, and 0.52 for anti-dsDNA.

**Table 2:** Results for anti-Rib-P, anti-Sm and anti-dsDNA quantification

	**SLE**	**HCs**		**RDCs**	
			*P*		*P*
Anti-Rib-P (U/ml)	4.9 ± 20.2	0.07 ± 0.21	0.016	0.6 ± 1.8	0.017
Anti-Rib-P(+), n (%)	18 (14.2%)	0 (0%)	<0.001	2 (0.8%)	<0.001
Anti-Sm (U/ml)	2.7 ± 13.8	0.02 ± 0.11	0.028	0.1 ± 0.3	0.035
Anti-Sm(+), n (%)	12 (9.4%)	0 (0%)	<0.001	0 (0%) )	<0.001
Anti-dsDNA (U/ml)	44.6 ± 73.8	3.5 ± 8.1	<0.001	2.6 ± 4.2	<0.001
Anti-dsDNA(+), n (%)	63 (49.6%)	6 (6.0%)	<0.001	5 (2.0%)	<0.001

Abbreviations: Anti-dsDNA, anti-double-stranded DNA; anti-Rib-P, anti-ribosomal P; anti-Sm, anti-Smith; SLE, systemic lupus erythematosus. Values represent mean ± standard deviation for concentrations in U/ml and proportions of positives. Differences were assessed using the *t*-test for continuous variables or the χ^2^ or Fisher’s exact test for proportions.

Only 2 samples (1.6%) were positive for all three tested autoantibodies, whereas 12 (9.4%) were positive for both anti-Rib-P and anti-dsDNA, and 7 (5.5%) were positive for both anti-Sm and anti-dsDNA. Cross-positivity for anti-Rib-P and anti-Sm was not seen (Table 3).

**Table 3:** Cross-positivity for the three determined autoantibodies (anti-Rib-P, anti-Sm, and anti-dsDNA) in patients with SLE

**Positive for:**	**n**	**%**
None	57	44.9
Anti-Rib-P only	4	3.1
Anti-Sm only	3	2.4
Anti-dsDNA only	42	33.1
Anti-Rib-P & Anti-Sm	0	0
Anti-Rib-P & Anti-dsDNA	12	9.4
Anti-Sm & Anti-dsDNA	7	5.5
All three autoantibodies	2	1.6

Abbreviations: Anti-dsDNA, anti-double-stranded DNA; anti-Rib-P, anti-ribosomal P; anti-Sm, anti-Smith; SLE, systemic lupus erythematosus.

The relationship between the clinical variables and the levels of anti-Rib-P, anti-Sm, and anti-dsDNA autoantibodies was further analyzed for the SLE group (Table 4).

**Table 4:** Clinical variables associated with anti-Rib-P, anti-Sm and anti-dsDNA levels in patients with SLE

**Variables**	**Anti-Rib-P**^**a**^		**Anti-Sm**^**b**^		**Anti-dsDNA**^**c**^	
	**β coefficient**	***P***	**β coefficient**	***P***	**β coefficient**	***P***
Ethnicity (Caucasian)	−0.190	0.034				
CRP, mg/dl			0.304	0.003		
Presence of serositis^d^			0.321	0.002		
Anti-RNP positive			0.297	0.003		
Disease duration, years					−0.246	0.005
SLEDAI2K					0.338	<0.001
Presence of renal disorder^d^					0.252	0.004

Abbreviations: Anti-dsDNA, anti-double-stranded DNA; anti-Rib-P, anti-ribosomal P; anti-RNP: anti-ribonucleoprotein; anti-Sm, anti-Smith; CRP: C-reactive protein; ESR: erythrocyte sedimentation rate; SLE, systemic lupus erythematosus; SLEDAI2K: Systemic Lupus Erythematosus Disease Activity Index 2000. Multivariate analysis results from multiple linear regression analysis. The total explained variance of the model was ^a^*R*^2^ *=* 0.036, ^b^*R*^2^ *=* 0.325, and ^c^*R*^2^ *=* 0.270. ^d^In accordance with American College of Rheumatology classification criteria.

Anti-Rib-P levels were related at (*P* ≤ 0.20) in univariate analysis with age (β *=* −0.125), Caucasian ethnicity (β *=* −0.190), erythrocyte sedimentation rate (ESR; β *=* 0.175), disease activity (SLEDAI2K; β *=* 0.154), malar rash (β *=* 0.142), renal disorder (β *=* 0.153), hematologic disorder (β *=* 0.130), and current corticosteroid dosage (β *=* 0.119). Hence, these variables were included in the multivariate analysis, which showed that Caucasian ethnicity (β *=* −0.190, *P =* 0.034) was the only factor independently associated with anti-Rib-P levels in patients with SLE (Table 4). Anti-Rib-P antibodies were not associated with previous neurologic disorder (seizure or psychosis) or with the occurrence of neuropsychiatric lupus features within the subsequent 3 years of follow-up.

The variables potentially associated with anti-Sm levels from the univariate analysis (at *P* ≤ 0.20) were Caucasian ethnicity (β *=* −0.060), ESR (β *=* 0.203), C-reactive protein (CRP) (β *=* 0.372), SLEDAI2K (β *=* 0.125), malar rash (β *=* −0.138), photosensitivity (β *=* 0.148), serositis (β *=* 0.277), renal disorder (β *=* 0.176), anti-RNP antibodies (β *=* 0.304), current corticosteroid dosage (β *=* 0.164), and use of immunosuppressants (β *=* 0.209). Higher CRP levels (β *=* 0.304, *P =* 0.003), serositis (β *=* 0.321; *P =* 0.002), and previous positivity for anti-RNP antibodies (β *=* 0.297; *P =* 0.003) were found to be independently associated with anti-Sm levels in patients with SLE (Table 4).

For anti-dsDNA levels, age (β *=* −0.237), age at disease onset (β *=* −0.169), disease duration (β *=* −0.176), ESR (β *=* 0.187), SLEDAI2K (β *=* 0.413), arthritis (β *=* −0.150), renal (β *=* 0.287), hematologic (β *=* 0.259), and immunologic disorders (β *=* 0.186), and current corticosteroid dosage (β *=* 0.130) came out as candidate predictors for higher anti-dsDNA levels (at *P* ≤ 0.20 in univariate analysis).In the multivariate analysis, SLEDAI2K (β *=* 0.338; *P* < 0.001), renal disorder (β *=* 0.252; *P =* 0.004), and shorter disease duration (β *=* −0.246; *P =* 0.005) were found to be independently associated with anti-dsDNA levels (Table 4).

## Discussion

Confirming earlier studies, the current work shows that anti-Rib-P protein autoantibodies are very specific for SLE diagnosis. The presence of antibodies against ribosomal P proteins was found to be very specific for patients with SLE compared with either HCs or with controls who had other rheumatic diseases. Moreover, the test had high levels of specificity and sensitivity. However, the choice of the most reliable test to determine these autoantibodies requires a comparative study between different tests and the study of a larger and multi-ethnic population.

In addition to determining the levels of anti-Rib-P autoantibodies, we used the same FEIA detection method to determine levels anti-Sm and anti-dsDNA autoantibodies in the same study groups. Both anti-Sm and anti-dsDNA antibodies have also been reported to be very specific for patients with SLE [21-23]; however, we found that anti-dsDNA antibodies were present at low levels in 6% of HCs and 2% of RDCs samples.

The commercial kit that we used for the determination of anti-Rib-P protein (EliA test) is an FEIA, designed as a sandwich immunoassay, containing a mixture of the three Rib-P antigens (P0, P1, and P2), which has been described previously as having high sensitivity and specificity [7,11,24]. We also used ROC curves to check the accuracy of this kit for the Portuguese population. ROC curves can be used to evaluate the diagnostic performance of a test, adjusting for a particular study population, and to determine the capability of a test to allow discrimination between the positive group and the control group [25,26]. Based on the ROC curves, we adjusted the cut-off values for both anti-Rib-P and anti-Sm to 4.45 U/ml and 3.4 U/ml, respectively. These values corresponded to the lowest concentration that allowed the highest possible sensitivity without losing specificity, establishing a cut-off value for the SLE group in comparison with the HC and RDC groups. For anti-dsDNA determination, we used the manufacturer’s cut-off value (15 U/ml) in subsequent analyses, as this gave the best combination of sensitivity and specificity. The cut-off confirmation should be performed when using a new kit or when using an existing kit in a different population. The adjusted values might be either higher or lower than those established by the manufacturer, as confirmed by the work of Mahler and colleagues [12].

Our results showed increased levels of all the three autoantibodies in patients with SLE, and a higher percentage of positive samples for at least one of the autoantibodies in the SLE group. Although anti-dsDNA autoantibodies were present in more individuals in the SLE group than in the other two groups, the presence of anti-Rib-P or anti-Sm was more specific for SLE diagnosis.

We reviewed the medical records of the individuals in the HC and RDC groups who had a positive result for either anti-Rib-P or anti-dsDNA antibodies (none was positive for anti-Sm). Both of the anti-Rib-P-positive results were detected in patients with RA, one of these patients had presented with some lupus-like characteristics (skin rash, leucopenia, and aphthous ulcer) at some point in the disease course, and thus this case could be classified as an overlap RA/SLE. Interestingly, a similar case was previously reported, referring to a anti-Rib-P-positive patient with RA, who later developed renal disease, and their condition evolved into full-blown SLE [11]. None of our HC or RDC group who were positive for anti-dsDNA autoantibodies had presented any lupus-like characteristics at any time.

When we used multivariate analysis on our SLE group, the only independent association with anti-Rib-P antibodies we identified was ethnicity: lower anti-Rib-P levels were present in individuals of Caucasian ethnicity. To our knowledge, no previous reports have established this association. However, given the small number of non-Caucasian patients in our group, these findings need to be replicated in larger SLE populations with different ethnic backgrounds.

Many previous studies have reported a relationship between the presence of anti-Rib-P antibodies and that of some clinical features, namely malar rash, renal involvement, and neuropsychiatric events, particularly psychosis [8,11,27,28]. However, there are also reports that corroborate our findings of an absence of such an association between the presence of anti-Rib-P antibodies and clinical features or disease activity [7,13,16]. In addition, our analysis differed from previous reports because we also took into consideration anti-Rib-P levels.

We found that Rib-P positivity was not associated with previous neuropsychiatric features classifiable by the ACR criteria [29,30], and the presence of these autoantibodies did not have a predictive value for the occurrence of neuropsychiatric symptoms in the subsequent 3 years. Thus, these autoantibodies seem to be very specific for SLE, but their value for diagnosis of neuropsychiatric lupus seems to be limited, possibly because both anti-Rib-P positivity and neuropsychiatric symptoms are relatively rare. However, given the high specificity, the inclusion of these autoantibodies as part of the SLE classification criteria might be useful. To confirm this, further studies encompassing a larger and multi-ethnic population are needed. Besides its possible use in established SLE, it will be important to assess the performance of such a test in patients with early-stage disease to confirm whether the inclusion of anti-Rib-P testing can improve diagnostic accuracy for SLE.

We performed a multivariate analysis for anti-Sm and anti-dsDNA levels, which revealed some associations of these antibodies with features of the disease. Serositis and CRP levels were positively associated with higher anti-Sm levels. High CRP levels are usually associated with an ongoing infection in patients with SLE, although they have been also associated with serositis, independently of the existence of an infection [31,32]. This is in line with our results, as we found that CRP levels were increased in patients with serositis (*P =* 0.047). However, the association between serositis and anti-Sm antibodies contradicts the results of a previous report from Wang and co-workers [33].

The multivariate analysis also showed anti-RNP levels to be independently associated with anti-Sm levels. Both anti-Sm and anti-RNP antibodies recognize complexes that contain small nuclear RNA species, and the occurrence of anti-Sm antibodies along with anti-RNP antibodies has been reported previously [34].

Our observations regarding anti-dsDNA are in line with other classic findings depicting an association with renal involvement, as well as in relation to lower disease duration and higher disease activity [35].

We also evaluated cross-positivity for the three studied autoantibodies, and verified that 78% of the anti-Rib-P-positive patients were also positive for one or both of the other antibodies determined. Previous studies have also shown that the presence of anti-Rib-P antibodies is often associated with anti-dsDNA antibodies, but the simultaneous presence of anti-Rib-P and anti-Sm is not consensual between studies [2,8,11,13]. However, we found that four patients (3.1%) in our SLE group (22% of the anti-Rib-P-positive patients) were positive for anti-Rib-P autoantibodies only. We reviewed the clinical records of these four patients, and found no particular clinical features in common.

## Conclusions

The presence of anti-Rib-P antibodies in patients negative for anti-DNA and anti-Sm indicates that these autoantibodies might be useful for SLE diagnosis, as previously reported by Mahler and colleagues [10]. Based on previous suggestions by other authors, and considering that disease classification criteria are constantly subject to confirmation and re-evaluation studies, as recently published by Petri and colleagues [36], we propose that further studies should be performed to evaluate the relevance of anti-Rib-P antibody testing for SLE diagnosis.

## Abbreviations

ACR: American College of Rheumatology; AS: Ankylosing spondylitis; AUC: Area under the curve; ESR: Erythrocyte sedimentation rate; CRP: C-reactive protein; FEIA: Fluorescence enzyme immunoassay; HC: Healthy control; IL: Interleukin; JIA: Juvenile idiopathic arthritis; LR: Likelihood ratio; PsA: Psoriatic arthritis; RA: Rheumatoid arthritis; RD: Rheumatic disease; ROC: Receiver operating characteristic; SLE: Systemic lupus erythematosus; SLEDAI2k: SLE Disease Activity Index 2000; SLICC: Systemic Lupus International Collaborating Clinics/ACR Damage Index; TNF: Tumor necrosis factor

## Competing interests

Laboratory expenses were supported by Phadia (now Thermo Fisher Scientific).

## Authors’ contributions

DCF was involved in the study design and performed the laboratory work, data analysis, and manuscript writing, MJS, HC, and JEF were involved in the study design; data acquisition, interpretation, and analysis; and critical revision of the article. All authors read and approved the final manuscript.

## Pre-publication history

The pre-publication history for this paper can be accessed here:

http://www.biomedcentral.com/1741-7015/11/98/prepub
